# Æsthetics of Medicine

**Published:** 1856-09

**Authors:** 


### *Æsthetics of Medicine.*

The science of medicine has enlarged its bounds in all directions. The microscope has opened to our view the secrets of minute anatomy; the patient experimenter tells of the varied functions of the organism of man; whilst chemistry, with rapid strides, has invaded the domain of vitalism, and thrown new and brilliant light on the darkest portions of the animal economy. During this period of rapid advance in the theory of medicine, the more practical departments of the art have not been stationary, and the changes and improvements in the management of disease, both medical and surgical, are even more wonderful than any of the discoveries of the micrographer or the experiments of the physiologist.

If the question could have been asked Sir Astley Cooper or old Ben Bell, what would be the greatest boon which could be vouchsafed the surgeon, he would have replied, "give me the power of operating without pain." It is done. In the language of an eloquent contemporary, "the knife is searching for disease; the pulleys are dragging back dislocated limbs; nature herself is working out the primal curse which doomed the tenderest of creatures to the sharpest of her trials; but the fierce extremity of suffering has been steeped in the waters of forgetfulness, and the deepest furrow in the knotted brow of agony has been smoothed forever.

The wondrous effects of ether are hardly known, before, in the discovery of chloroform, we have a pain controller of even greater power, whilst the class of "local anæsthetics" is increasing daily, and now we read of tumors painlessly removed under the influence of the freezing mixture, and the still more recently discovered properties of carbonic acid gas.

These triumphs of art have almost blinded us to the many improvements which chemistry and pharmacy are constantly yielding in the modified and improved preparations and combinations of medicine, and in admiring the showy and striking results of anæsthesia in the field of surgery, the more humble but yet equally valuable discoveries in the department of medicine are in danger of being overlooked. Medicine has its æsthetics as well as surgery, and the sick chamber, under the influence of the refinements of pharmacy is rapidly losing much of its ancient horrors.

Compare the condition of a sufferer wasting away with slow fever, or some other prolonged and painfull malady, as he now is, and as he once was. Then, the curtained bed and the carefully closed room guarded his fevered body from the slightest breath of air. Now, the ventilated chamber, the open window, the grateful fan and the cheerful light are kindly and liberally vouchsafed him. Then, the cautious doctor warned him of the danger of one mouthful of water. Now, the tinkling ice and the refreshing acid cools his parching throat. The nauseated stomach was then thrown into increased torture with neutral draughts and saline mixtures; but now the citrate of potass delights the palate and calms the unnatural temperatures, and "Abernethy's black bottle" is replaced with the effervescing magnesia, which charms the fastidious taste, and allays the irritable organs.

But let us imagine our unfortunate to have survived the rude shocks of pill and portion, and only needing the cordial tonic to bring him at last from his chamber of torture. He is not yet released. Bark, says his attendant, is the one thing needful, and hourly doses of the ligneous material distend his stomach, intermixed it may be with the still more revolting electuary, whose nauseating sweets make the whole mass a perfection of horrors. How different is the fate of him whose happy lot has fallen in these æsthetic days. His speck of crystallized quinine is impenetrably concealed in the fragrant coffee, or neutralized with tannin, and his iron and treacle transformed into the elegant formulæ of Blancard or Vallet. Should his ingenious and complaisant medical adviser be sometimes forced to appeal to some of the old physics, he calls for a capsule of Lehuby or Requin, and envelops in its gelatinous cavity the once dreaded remedy.

Such are some of the triumphs of pharmacy over the armamentum medicum of our fathers; and let not our readers disregard the great advantages to be derived from their use in the management of disease. Under their influence, we can often make the sick bed a place of comparative comfort, and even use our remedies alike to gratify and to cure. In the treatment of children, these elegant preparations are of inconceivable value. No longer does the little victim's nose writhe between the thumb and fore finger of his determined nurse, or the medicine spoon force its way between the resisting teeth; but now the patient eagerly looks for his medicine, and hugs his once dreaded foe to his breast. The physician is thus enabled to cultivate with the children under his charge the most kindly relations, and the hour of visit, which was once the signal for discord, is now transformed into a friendly reunion.—*Virginia Med. Journal.*

